# The histone methyltransferase Suv39h regulates 3T3-L1 adipogenesis

**DOI:** 10.1080/21623945.2020.1795422

**Published:** 2020-07-22

**Authors:** Jia Jing, Fenfen Li, Lin Zha, Xiaosong Yang, Rui Wu, Shirong Wang, Bingzhong Xue, Hang Shi

**Affiliations:** a Department of Biology, Georgia State University, Atlanta, GA, USA; b Clinical Center of Spaceport, Chinese PLA General Hospital, Beijing, China; c Key Laboratory on Cardiovascular, Cerebrovascular, and Metabolic Disorders, Hubei University of Science and Technology, Xianning, China

**Keywords:** Suv39h, histone methylation, epigenetics, adipogenesis, adipocyte

## Abstract

We discovered a unique expression pattern of two histone methyltransferases Suv39h1 and Suv39h2 during 3T3-L1 adipogenesis, both of which preferentially catalyse the formation of H3K9 dimethylation (H3K9me2) and further H3K9 trimethylation (H3K9me3), a transcriptional repressive mark. The expression of Suv39h1 and Suv39h2 displayed a sharp increase at the early stage of 3T3-L1 differentiation, which peaked after differentiation induction, and then declined towards later stage of differentiation, suggesting a key role for these two histone methyltransferases in adipogenesis. Indeed, inactivating Suv39h1 or Suv39h2 via lentiviral shRNA knockdown inhibited adipogenesis, while overexpressing Suv39h1 promoted adipogenesis. Notably, overexpressing or knocking down Suv39h1 in 3T3-L1 cells was associated with reciprocal changes in the expression of Wnt10a, an anti-adipogenic regulator. Further, Wnt10a knockdown largely prevented the inhibitory effect of Suv39h1 on adipogenesis, indicating Wnt10a as a downstream target mediating Suv39h1’s action in adipogenesis. Mechanistically, our comprehensive approaches involving ChIP, co-immunoprecipitation and pyrosequencing analysis demonstrated that Suv39h1 may regulate Wnt10a expression via H3K9 methylation and interaction with DNA methyltransferase 1 (DNMT1) at the Wnt10a promoter, resulting in altered DNA methylation at the promoter. We conclude that Suv39h promotes adipogenesis by epigenetically down-regulating Wnt10a expression via H3K9me3 and DNA methylation at the Wnt10a promoter.**Abbreviated title:** Suv39h and 3T3-L1 Adipogenesis

## Introduction

Obesity is characterized by increased white adipose tissue mass, which expands by adipocyte hypertrophy and hyperplasia [1,2]. Better understanding the molecular mechanisms of adipogenesis, which is mainly responsible for adipocyte formation or hyperplasia, may provide potential therapeutic approaches to limit adipose expansion and thereby treat obesity. Regulation of adipogenesis has been extensively investigated and entails a sequential activation of key transcriptional factors along the course of differentiation, including members of CCAAT/enhancer-binding protein (C/EBP) family, namely, C/EBPα, β and δ, and the nuclear receptor peroxisome proliferator γ (PPARγ) [3]. Adipogenesis begins with an induction of C/EBPβ and δ at the early stage of differentiation. The two transcriptional factors further induce the expression of PPARγ and C/EBPα, interaction of which later leads to commitment of the preadipocytes to the mature adipocytes by inducing a panel of adipocyte phenotypic genes involved in glucose and lipid metabolism [3]. On the other hand, a number of transcriptional repressors and anti-adipogenic factors, such as GATA-binding proteins (GATA2/3), chicken ovalbumin upstream promoter transcription factor (COUP-TF), interferon regulatory factors (IRFs), and the wingless-type MMTV integration site (WNT) family proteins, act to maintain pre-adipocyte or precursor cell stage by silencing the adipogenic process through counter-regulating the pro-adipogenic transcriptional factors [3–7]. Adipogenesis that determines the fate of preadipocytes is tightly regulated by a coordinated control of the pro- and anti-adipogenic factors.

While transcriptional regulation of adipogenesis has been extensively investigated and well documented, much less is known about the epigenetic pathways underlying this process. Epigenetic regulation, which modulates gene expression through organizing chromatin structure at different hierarchical levels via covalent modifications on histones (e.g. lysine methylation and acetylation) or DNA (e.g. cytosine methylation at CpG dinucleotides), has emerged as a key link between environmental factors (e.g. diets) and metabolic disorders (e.g. obesity) [8–11]. Histone methylation is made by a transfer of up to three methyl groups from S-adenosylmethionine to generate mono-, di-, or tri-methylation at the lysine residues of the histones [12]. Histone lysine methylation mainly occurs to histone H3 at multiple lysine sites (H3K4, H3K9, H3K27, H3K36, and H3K79) and to histone H4 at a single site (H4K20) [12]. Histone methylation can result in either gene activation or gene repression, depending on the specific position of the lysine and the level of the methylation (mono-, di-, or tri-methylation) [12]. While trimethylated H3K4 (H3K4me3) and H3K36 (H3K36me3) are often associated with transcriptional active genes, trimethylated H3K9 (H3K9me3) and H3K27 (H3K27me3) are a hallmarks of gene silencing [12]. Histone methylation levels are tightly controlled by a regulatory system that consists of site-specific histone methyltransferases, demethylases and methyl recognition proteins, which play key roles in the regulation of gene expression via altering chromatin structure and interacting with other transcriptional proteins [12].

To study whether epigenetic regulation plays a role in adipogenesis, we surveyed the expression of most epigenetic enzymes that catalyse histone methylation and acetylation in the course of 3T3-L1 adipocyte differentiation. We identified a unique expression pattern of two histone methyltransferases suppressor of variegation 3–9 1 (Suv39h1) and 2 (Suv39h2), both of which preferentially catalyse the formation of H3K9 dimethylation (H3K9me2) or further H3K9 trimethylation (H3K9me3), a transcriptional repressive mark. Using gain or loss of function approaches, we activated or inactivated SVU39H enzymes and examined adipogenesis by measuring the expression of adipogenic markers and staining cellular lipid accumulation with Oil Red O. In an attempt to explore the molecular mechanisms underlying the changes of the adipogenic phenotypes, we screened a number of transcriptional repressors and identified the anti-adipogenic factor Wnt10a as a potential mediator. We further discovered that Suv39h may promote adipogenesis by down-regulating Wnt10a expression via H3K9me3 and DNA methylation at the Wnt10a promoter.

## Materials and methods

### 3T3-L1 preadipocyte culture and differentiation

3T3-L1 preadipocytes were cultured and differentiated as we previously described [13,14]. Briefly, 3T3-L1 preadipocytes were cultured in a DMEM growth medium containing 10% new born calf serum (NBCS) and 1% Penicillin/Streptomycin antibiotics. Confluent preadipocytes were induced to differentiate with an adipocyte differentiation medium containing 10% foetal bovine serum (FBS), 0.5 mM 3-isobutyl-1-methylxanthine (IBMX), 1 μM dexamethasone, 400 nM insulin and antibiotics. Cells were cultured with the differentiation medium for 2 days and then changed to a DMEM maintenance medium containing 10% FBS, 400 nM insulin and antibiotics for another 2 days. Afterwards, cells were maintained in a DMEM medium containing 10% FBS and antibiotics throughout the adipocyte stage and culture medium was changed every 2 days.

### Generation of the 3T3-L1 stable cell lines with Suv39h1 or Suv39h2 knockdown

The Suv39h1 and h2 lentiviral shRNA constructs were purchased from Dharmacon (Lafayette, CO). The Suv39h shRNA lentivirus was generated as we previously described [14,15]. Briefly, the Suv39h lentiviral vectors or control vectors were co-transfected with the viral packaging plasmids pCMV-dR8.91 and the envelope plasmids VSV-G (both kindly provided by Dr. Evan Rosen from Beth Israel Deaconess Medical Centre) into 293 T cells. The lentivirus-containing medium was harvested and filtered, and was used to infect 3T3-L1 preadipocytes. The infected cells underwent puromycin (2 µg/ml) selection for 5 days, and all the surviving cells were pooled to generate the 3T3-L1 stable cell lines with Suv39h knockdown.

### SiRNA knockdown of Wnt10a

The ON-TARGET plus Mouse Wnt10a SiRNA-SMART pool was purchased from Dharmacon and SiRNA knockdown of Wnt10a in Suv39h-knockdown stable cells was performed as we previously described [14,16,17]. Briefly, the 3T3-L1 stable cells with Suv39h knockdown were reversely transfected with Wnt10a SiRNA to achieve gene knockdown using Lipofectamine RNAiMAX Reagent (ThermoFisher Scientific, Waltham, MA). The cells were induced for adipocyte differentiation 2 days after.

### Generation of the 3T3-L1 stable cell line with Suv39h1 over-expression

The full length Suv39h1 cDNA was purchased from Dharmacon and further subcloned into the pLVX lentiviral expression vector (Clontech). Lentivral production, infection of 3T3-L1 preadipocytes, and establishment of stable cells overexpressing Suv39h1 were conducted as described above.

### Suv39h1 and DNMT1 expression constructs

The full length cDNA of Suv39h1 or DNMT1 was purchased from Dharmacon and further subcloned into the pcDNA3.1 expression plasmid.

### Total RNA extraction and quantitative RT-PCR

Total RNA was extracted from the cells using the Tri Reagent kit (Molecular Research Centre, Cincinnati, OH), according to the manufacturer’s protocol. The expression of adipogenic genes was measured by a one-step quantitative RT-PCR with TaqMan Universal PCR Master Mix reagents (Applied Biosystems. ThermoFisher) using a Stratagene Mx3005p thermocycler (Stratagene, La Jolla, CA), as we previously described [14,15,18]. The primer and probe pairs used in the assays were purchased from Applied Biosystems (ThermoFisher). The mRNA quantitation was further normalized by the housekeeping gene cyclophilin.

### Immunoprecipitation (IP) and immunoblotting (IB)

IP and IB were performed as we previously described [14,16,17]. Briefly, cells were harvested and homogenized in a modified radioimmunoprecipitation assay (RIPA) lysis buffer containing 50 mM Tris-HCl, 1 mM EDTA, 1% Nonidet P-40, 0.25% sodium deoxycholate, 150 mM NaCl, 1 mM phenylmethylsulfonyl fluoride, 200 µM Na3VO3, 1% protease inhibitor mixture (Sigma, St. Louis, MO), and 1% phosphatase inhibitor mixture (Sigma). Cell homogenates were incubated on ice for 45 min to solubilize all proteins, and followed by centrifugation at 14,000 *g* at 4°C for 15 min. 1 mg cell lysates were incubated with an anti-DNMT1 antibody (ab87656, Abcam, Cambridge, MA) and protein A/G agarose beads (Sigma-Aldrich) at 4°C overnight. The beads were washed, boiled in Laemmli sample buffer and used for SDS-PAGE. For IB, 50 µg of cell lysates were boiled in Laemmli sample buffer and used for SDS-PAGE. The gels were transferred to nitrocellulose membrane (Bio-Rad, Hercules, CA). The transferred membranes were blocked, washed, and incubated with various primary antibodies, followed by Alexa Fluor 680-conjugated secondary antibodies (Life Science Technologies). The blots were developed with a Li-COR Imager System (Li-COR Biosciences, Lincoln, NE). The antibodies are anti-PPARγ antibody (sc-7196, Santa Cruz, CA), anti-CEBPα antibody (MA1-825) and anti-GAPDH antibody (MA5-15,738) (ThermoFisher), anti-Suv39h1 antibody (D11B6, 8729, Cell Signalling), anti-Suv39h2 antibody (ab190870, Abcam).

### Chromatin immunoprecipitation (ChIP) assay

ChIP assays were performed using a ChIP assay kit (17–295, Millipore, Temecula, CA) as we previously described [16–18]. Briefly, cells were fixed with 1% of formaldehyde and then harvested in cell lysis buffer (5 mM PIPES, 85 mM KCl, and 0.5% NP-40, supplemented with protease inhibitors, pH 8.0). Genomic DNA in lysates were sheared into fragments with an average length of 200–1,000 bp using a programmed sonication machine (Bioruptor, Diagenode, Denville, NJ). Lysates were further centrifuged to collect the supernatants that contain the sheared DNA, which underwent overnight immunoprecipitation, elution, reverse cross-linking, and protease K digestion. The DNAs recovered from phenol/chloroform extraction were used for SYBR Green quantitative PCR (Applied Biosystems) and was further normalized to the DNA quantitation of individual input control. The ChIP antibody against H3K9me3 (ab8898) and DNMT1 (ab87656) was purchased from Abcam (Cambridge, MA). The sequences of primers for the *Wnt10a* promoter are: Forward: 5ʹ-CATGAGGTCCGTGGGTTTGTA-3ʹ; Reverse: 5ʹ- CCTACACTGAAAACAGCGTGC-3ʹ.

### Pyrosequencing analysis of the Wnt10a promoter

The pyrosequencing analysis was conducted as we previously described [14,19]. Briefly, 3T3-L1 adipocyte genomic DNA was isolated by phenol-chloroform extraction. An EpiTech Bisulphite Kit (Qiagen, Valencia, CA) was used to conduct Bisulphite conversion on the CpG sites. The bisulphite-converted DNA was then used as template to amplify the DNA region covering the CpG sites at the Wnt10a promoter and followed by the pyrosequencing that was commercially provided by EpiGenDx (Hopkinton, MA). The PCR and pyrosequencing primers that were used to measure DNA methylation levels on Wnt10a 5ʹ region are described in our previous publications [13,14] and in Supplemental Table 1.

### Oil Red O staining

Lipid accumulation in adipocytes during adipogenesis was assessed by Oil Red O staining as we previously described [13]. The Oil Red O compound was purchased from Sigma-Aldrich (St. Louis, MO) and was dissolved in isopropanol to make a 0.5% solution. Adipocytes were fixed with 10% formaldehyde for 1 h and washed before being stained with Oil Red O working solution for 10 minutes.

### Statistics

All data are expressed as mean±SEM. Data were evaluated for statistical significance by *t*-test, one-way ANOVA followed by Bonferroni’s post hoc test as appropriate, and statistical significance for comparison of means of different groups was calculated by using GraphPad Prism version 5.0. *p* < 0.05 was considered significant.

## Results

### The expression of Suv39h1/2 is increased in the early stage of adipogenesis

**Figure 1.**
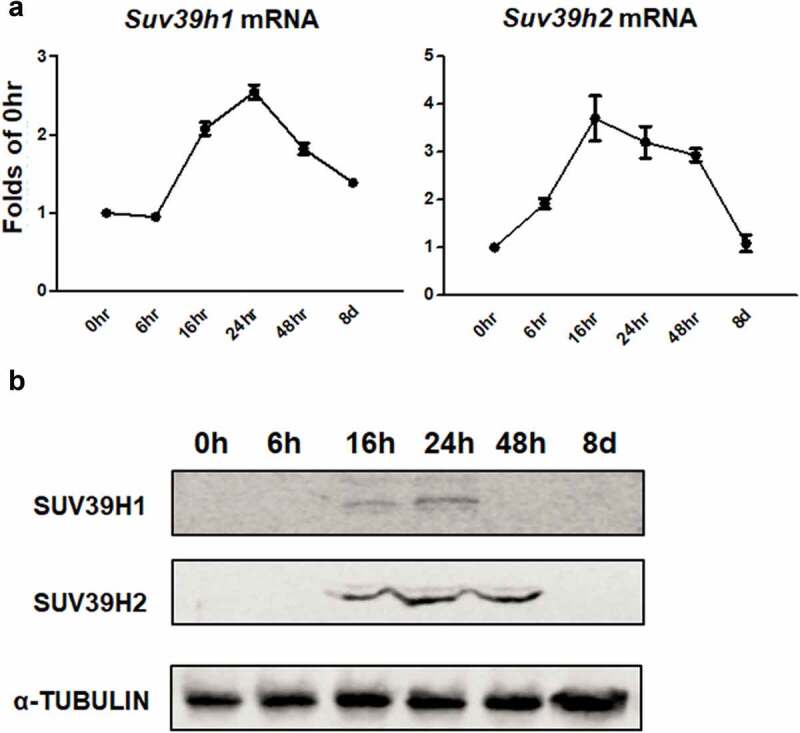
*Suv39h1/h2* expression is transiently induced during the early stage of 3T3-L1 adipogenesis. 3T3-L1 preadipocytes were induced to differentiation and cells were harvested at the time point indicated. *Suv39h1/h2* mRNA (a) and protein (b) levels were measured by quantitative PCR analysis and immunoblotting, respectively. All data are expressed as mean ± SEM, *n* = 4

To study whether epigenetic regulation plays a role in adipogenesis, we surveyed the expression of most epigenetic enzymes (e.g. histone methyltransferases, demethylases, etc.) that catalyse histone methylation and acetylation in the course of 3T3-L1 preadipocyte differentiation. For the preliminary screening, we pooled 4 RNA samples from each time point along the differentiation time course for the quantitative RT PCR measurement. Among the six genes encoding histone methyltransferases (Suv39h1, Suv39h2, G9a, and Eset) and demethylases (Kdm3a and Kdm4a) responsible for maintaining methylation of the histone repressive mark H3K9, we discovered a unique expression pattern of two histone methyltransferases Suv39h1 and Suv39h2 (Suppl. Figure 1a and 1b). We then further confirmed the expression pattern of Suv39h1 and Suv39h2 by quantitative PCR using four individual RNA samples (Figure 1(a)). We found that the mRNA levels of Suv39h1 and Suv39h2 were rapidly induced at the early stage of differentiation to reach peak levels at 16–24 hours and then gradually declined to the baseline levels at day 8 of the differentiation (Figure 1(a)). Similar results were observed in Suv39h1/h2 protein levels during the adipogenic time course (Figure 1(b)). The first 48 hours of differentiation represent a critical time window of adipogenesis, in which clonal expansion occurs and leads to irreversible progression towards the terminal fate of adipocytes [20]. The rapid induction of Suv39h1 and Suv39h2 suggests that these two methyltransferases may play critical roles in adipogenesis.

### Suv39h knockdown inhibits adipogenic gene expression

**Figure 2.**
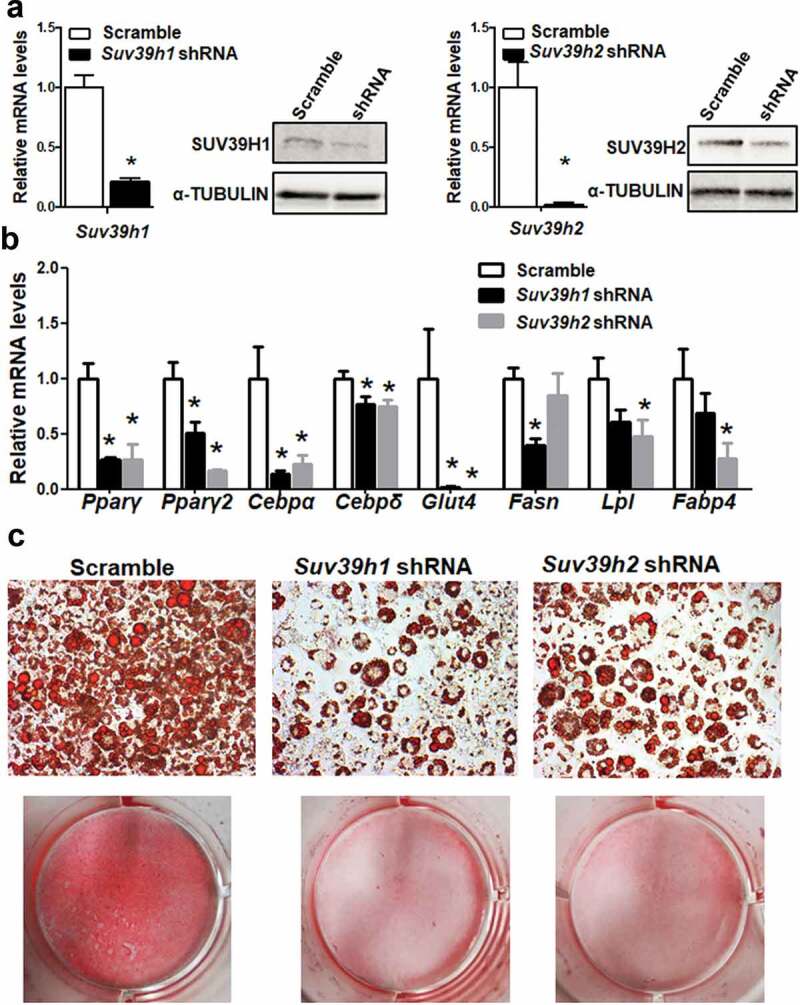
*Suv39h1* or *Suv39h2* knockdown suppresses 3T3-L1 adipogenesis. (a) *Suv39h1* or *Suv39h2* knockdown inhibits mRNA and protein levels of *Suv39h1* or *Suv39h2* in the 3T3-L1 cells. (b) *Suv39h1* or *h2* knockdown inhibits adipogenic gene expression in 3T3-L1 preadipocytes at day 4 of differentiation. (c) Oil Red O staining reveals less lipid accumulation in *Suv39h1* or*h2* knockdown cells at day 8 of differentiation. Microscopic image (upper) and whole well view (lower). 3T3-L1 preadipocytes were infected with *Suv39h1* or *h2* shRNA lentivirus, selected with puromycin and differentiated as described in the Methods. Gene expression was measured by quantitative PCR. All data are expressed as mean ± SEM, *n* = 4. *p < 0.05 vs. scramble control

**Figure 3.**
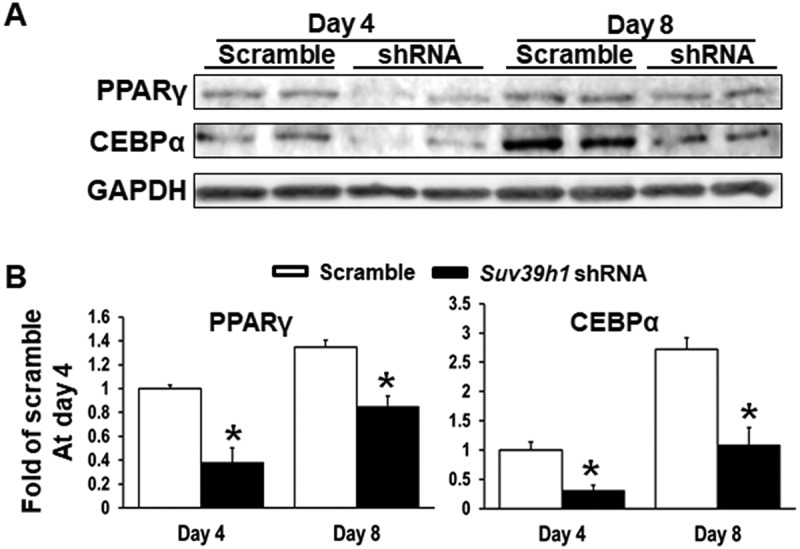
*Suv39h1* knockdown inhibits adipogenic protein expression in 3T3-L1 preadipocytes. (a) Immunoblots of PPARγ and CEBPα protein. (b) Density quantitation of the immunoblots. 3T3-L1 preadipocytes were infected with *Suv39h1* shRNA lentivirus, selected with puromycin and differentiated as described in the Methods. Protein levels were measured by immunoblotting. All data are expressed as mean ± SEM, *n* = 4. *p < 0.05 vs. scramble control

To study the functional role of *Suv39h* in regulation of adipocyte differentiation, we established 3T3-L1 preadipocyte cell lines with reduced *Suv39h1* or *Suv39h2* expression by lentiviral shRNA knockdown. 3T3-L1 cells infected with lentivirus expressing *Suv39h1* or *Suv39h2* shRNA exhibited a decrease in the mRNA and protein levels of *Suv39h1* and *Suv39h2* respectively (Figure 2(a)). While *Suv39h2* knockdown slightly increased *Suv39h1* mRNA expression, *Suv39h1* knockdown did not affect *Suv39h2* expression (Suppl. Figure 2). The 3T3-L1 preadipocytes with *Suv39h1* or *Suv39h2* knockdown were then induced to differentiate with the differentiation protocol. Eight days after induction of differentiation, cells were harvested for adipogenesis analysis. We found that inactivation of *Suv39h1* in 3T3-L1 adipocytes suppressed the expression of adipogenic genes such as *Pparγ, Cebpα*, and *Cebpδ*, and adipocyte phenotypic genes such as fatty acid synthase (*Fasn*) and glucose transporter 4 (*Glut4*) (Figure 2(b)). Similar results were observed in L1 cells with *Suv39h2* knockdown, which similarly exhibited decreased expression of genes involved in differentiation such as *Pparγ, Cebpα*, and *Cebpδ* and genes responsible for glucose and lipid metabolism such as *Glut4*, fatty acid binding protein 4 /422 adipose P2 protein (*Fabp4*/aP2) and lipoprotein lipase (*Lpl*) (Figure 2(b)). Our Oil Red O staining analysis revealed less lipid accumulation at day 8 of differentiation in *Suv39h1* and *Suv39h2* knockdown cells, further confirming the inhibition of adipogenesis by *Suv39h* deficiency in preadipocytes (Figure 2(c)). We also found decreased PPARγ and CEBPα protein levels in *Suv39h1* knockdown cells at day 4 and day 8 of the differentiation (Figure 3).

### Suv39h1 overexpression promotes adipogenic gene expression

**Figure 4.**
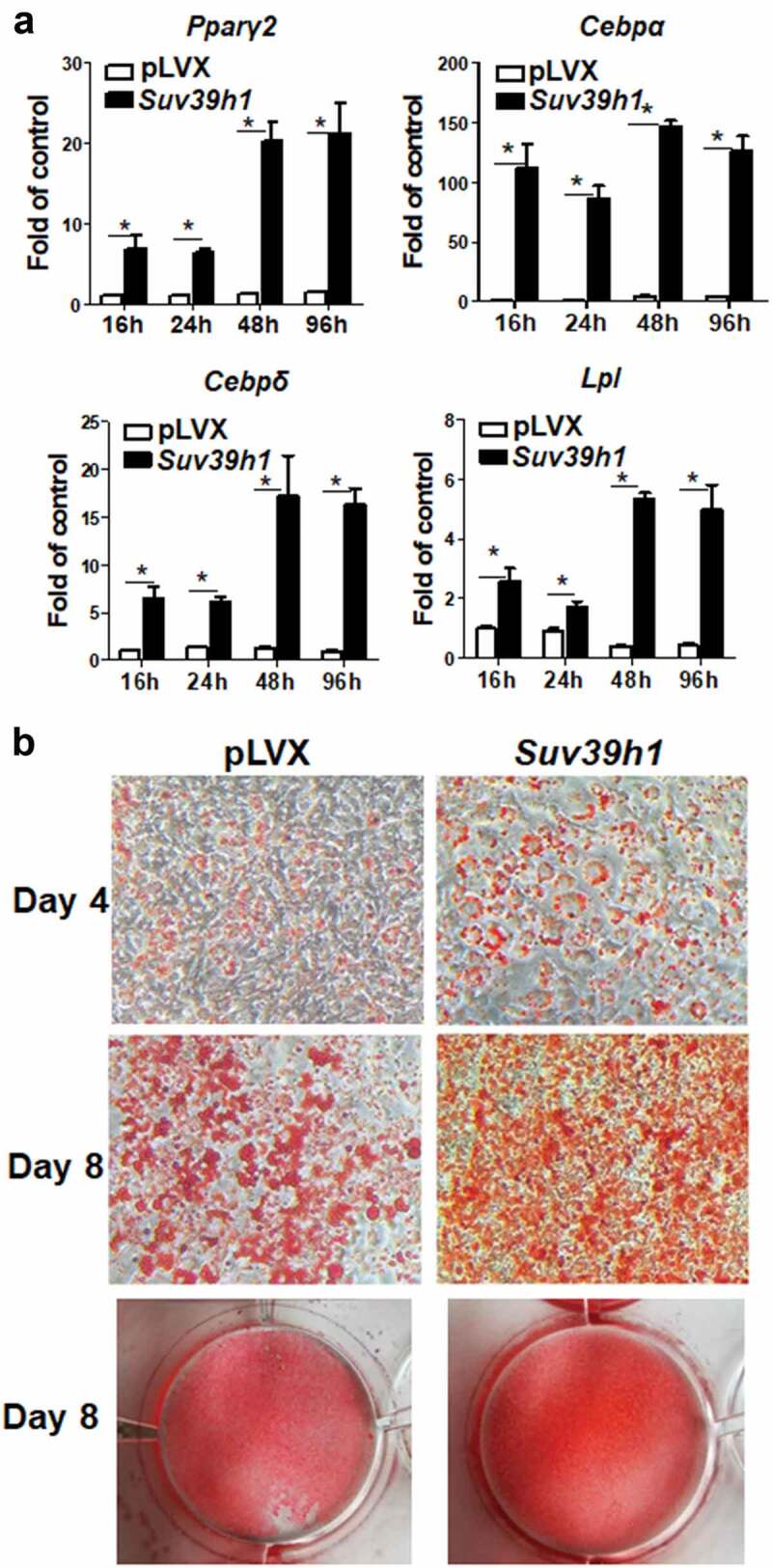
*Suv39h1* overexpression promotes 3T3-L1 adipogenesis. (a) *Suv39h1* overexpression promotes adipogenic gene expression in 3T3-L1 preadipocytes. (b) Oil Red O staining reveals more lipid accumulation in *Suv39h1* overexpressing cells. 3T3-L1 preadipocytes were infected with the *Suv39h1* pLVX lentivirus, selected with puromycin and differentiated as described in the Methods. Gene expression was measured by quantitative PCR. All data are expressed as mean ± SEM, *n* = 4. *p < 0.05 vs. scramble control

To study whether *Suv39h* is sufficient to promote adipogenesis, we employed a gain-of-function approach to over-express *Suv39h1* in 3T3-L1 preadipocytes, because *Suv39h1* and *Suv39h2* are similar in catalysing the tri-methylation of H3K9 [21] and because we found that *Suv39h1* knockdown in 3T3-L1 cells had a more profound effect on adipocyte differentiation as evident by the Oil Red O staining (Figure 2(c)) and that *Suv39h1* mRNA (Ct value 26.33/20 ng total RNA) appeared to be 3-cycle more abundant than that of *Suv39h2* (Ct value 29.22/20 ng total RNA) (Suppl. Figure 3). We established a L1 cell line with *Suv39h1* overexpression via lentiviral infection and puromycin selection. We confirmed the overexpression of *Suv39h1* mRNA and protein by quantitative PCR and immunoblotting, respectively (Suppl. Figure 4). Over-expression of *Suv39h1* in L1 preadipocytes promoted the expression of adipogenic markers such as *Pparγ, Cebpα, Cebpδ*, and *Lpl* along the course of differentiation spanning from 16 h to 4 days (Figure 4(a)). This was further confirmed by Oil Red O staining showing enhanced lipid accumulation in *Suv39h1*-overexpressing cells at day 4 and day 8 of differentiation (Figure 4(b)). These data suggest that *Suv39h1* is a positive regulator of 3T3-L1 adipogenesis.

### Wnt10a is a target for Suv39h1 in adipogenesis

**Figure 5.**
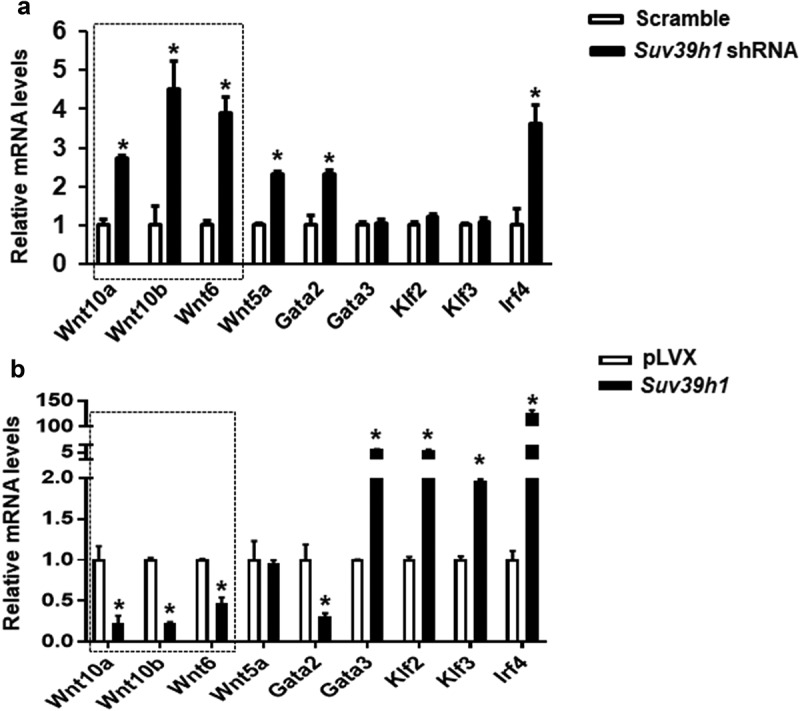
*Wnt* gene expression is reciprocally regulated by gain-or-loss of function of *Suv39h1*. The expression of three *Wnt* family members *Wnt10a, Wnt10b* and *Wnt6* was significantly up-regulated in *Suv39 h1* knockdown cells (a) while reciprocally down-regulated in *Suv39 h1*-overexpressing cells (b). 3T3-L1 preadipocytes were infected with *Suv39h1* shRNA lentivirus or pLVX *Suv39h1* expression lentivirus, selected with puromycin and differentiated as described in the Methods. Gene expression was measured by quantitative PCR. All data are expressed as mean ± SEM, *n* = 4. *p < 0.05 vs. scramble control or pLVX control

**Figure 6.**
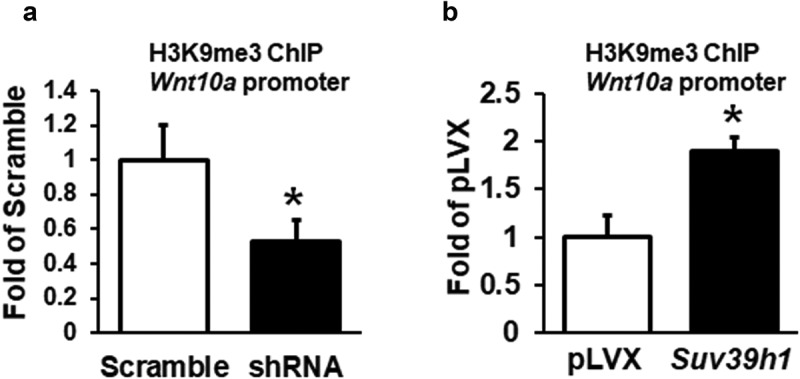
*Suv39h1* regulates tri-methylated H3K9 (H3K9me3) at the *Wnt10a* promoter. *Suv39h1* knockdown decreased (a), while *Suv39h1* overexpression increased (b) H3K9me3 levels at the *Wnt10a* promoter in 3T3-L1 preadipocytes. 3T3-L1 preadipocytes were infected with the *Suv39h1* or *h2* shRNA lentivirus, selected with puromycin and differentiated as described in the Methods. H3K9me3 at the *Wnt10a* promoter was assessed by ChIP assays with an anti-H3K9me3 antibody. All data are expressed as mean ± SEM, *n* = 3. *p < 0.05 vs. scramble control

**Figure 7.**
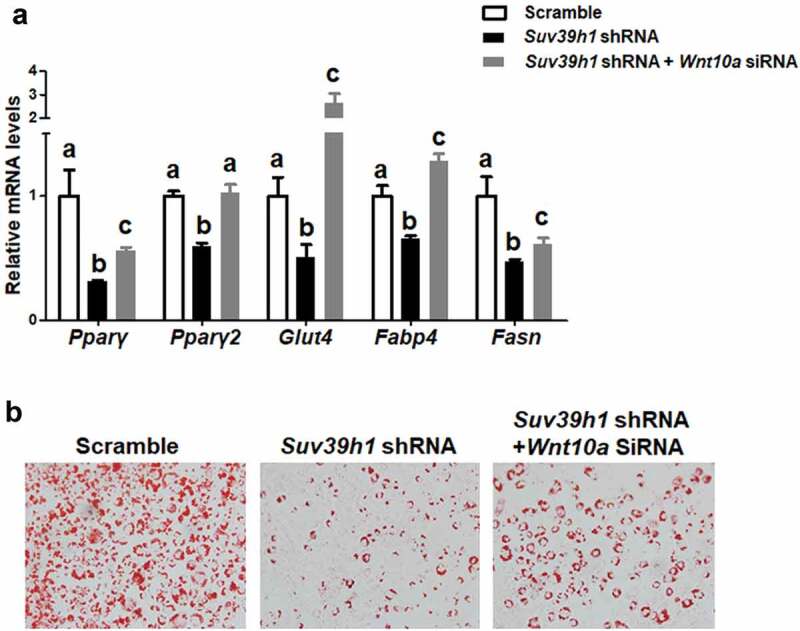
*Wnt10a* is a mediator of Suv39 h1’s action in adipogenesis. (a) *Wnt10a* knockdown substantially blocked the inhibitory effect of *Suv39h1* deficiency in the expression of adipogenic markers. *Suv39h1* knockdown preadipocytes were reversely transfected with *Wnt10a* siRNA and then differentiated into adipocytes as described in the Methods. Gene expression was measured by quantitative PCR. All data are expressed as mean ± SEM, *n* = 4. Bars labelled with different letters are statistically different from each other. (b) Oil Red O staining reveals increased lipid accumulation in *Suv39 h1/Wnt10a* double knockdown cells

To further explore the mechanism by which *Suv39h1* regulates adipogenesis, we primarily focused on anti-adipogenic factors based on a plausible assumption that *Suv39h*, a positive regulator of adipogenesis as shown above, would increase H3K9me3, a transcriptional repressive mark that is expected to cause the silencing of anti-adipogenic genes. To narrow down the anti-adipogenic factors that potentially mediate Suv39h’s effect, we surveyed the expression of several anti-adipogenic factors in both *Suv39h1* knockdown and overexpression L1 cells in a hope that genes as such with the reciprocal changes of their expression can be converged. Among the adipogenic repressors we measured, the expression of three WNT family members *Wnt10a, Wnt10b* and *Wnt6* was significantly up-regulated in *Suv39h1* knockdown cells while reciprocally down-regulated in *Suv39h1*-overexpressing cells (Figure 5(a,b)). Given the important role of *Wnt10a* in adipogenesis and our prior finding that *Wnt10a* is epigenetically regulated in adipogenesis [13], we interrogated whether *Wnt10a* is a downstream target of *Suv39h* in the regulation of adipogenesis. Trimethylated H3K9 (H3K9me3), whose formation is preferentially catalysed by *Suv39h*, is a hallmark of gene repression [12]. We therefore assessed the levels of H3K9me3 at the *Wnt10a* promoter in both *Suv39h1* knockdown and overexpression cells. We found that inactivation of *Suv39h1* by shRNA knockdown down-regulated H3K9me3 levels at the *Wnt10a* promoter while overexpression of *Suv39h1* did the opposite (Figure 6), which may explain the reciprocal change of *Wnt10a* mRNA in these cells. To further study whether *Wnt10a* is necessary in mediating *Suv39h*’s effect on adipogenesis, we knocked down *Wnt10a* in *Suv39h1* knockdown preadipocytes using siRNA approach (Suppl. Figure 5). We found that inactivation of *Wnt10a* substantially prevented the inhibitory effect of *Suv39h1* deficiency on the expression of adipocyte markers such as Pparγ, Glut4, *Fabp4*/aP2 and *Fasn* (Figure 7(a)). Further Oil Red O staining indicated that double knockdown of *Suv39h1* and *Wnt10a* substantially increased lipid-laden mature adipocytes compared to *Suv39h1* knockdown alone (Figure 7(b)), suggesting *Wnt10a* as a key mediator of *Suv39h1*’s action in adipogenesis.

### Wnt10a is epigenetically regulated by Suv39h1

**Figure 8.**
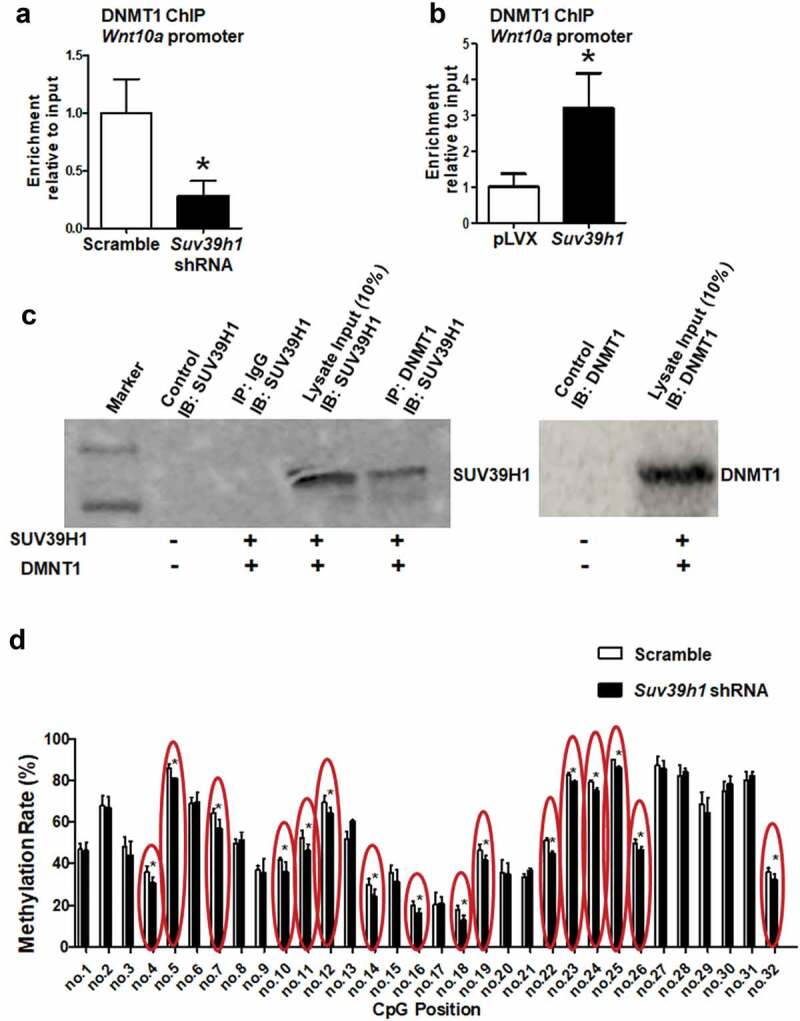
H3K9 methylation status affects recruitment of DNMT1 to the *Wnt10a* promoter. *Suv39h1* knockdown decreased (a), while *Suv39h1* overexpression (b) increased DNMT1 recruitment at the *Wnt10a* promoter in 3T3-L1 preadipocytes. 3T3-L1 preadipocytes were infected with *Suv39 h1* shRNA lentivirus or overexpression lentivirus, selected with puromycin and differentiated as described in the Methods. DNMT1 binding to the *Wnt10a* promoter was assessed by ChIP assays with an anti-DNMT1 antibody. All data are expressed as mean ± SEM. *p < 0.05 vs. scramble control or pLVX control. (c) Immunoprecipitation of DNMT1 pulls down the SUV39 H1 protein. Immunoprecipitation and immunoblotting were conducted as described in the Methods. 10% Input: without IP, SUV39H1 (left blot) or DNMT1 (right blot) immunoblotting only. (d) *Suv39h1* knockdown decreases CpG site methylation rate at the *Wnt10a* promoter. CpG methylation was measured by pyrosequencing analysis as described in the Methods. All data are expressed as mean ± SEM. *p < 0.05 vs. scramble control

It is known that there exists a close crosstalk between DNA methylation and histone methylation, which may act cooperatively to influence gene expression [22–24]. Our prior studies showed that DNA methylation of the *Wnt10a* promoter by DNA methyltransferase 1 (DNMT1) plays an important role in adipogenesis [13,14]. We next tested whether the change of H3K9 methylation would affect recruitment of DNMT1 to the *Wnt10a* promoter. Indeed, our ChIP assays showed that *Suv39h1* deficiency decreased DNMT1 binding to the *Wnt10a* promoter while *Suv39h1* overexpression did the opposite (Figure 8(a,b)), suggesting that DNMT1 may also be recruited to the *Wnt10a* promoter to silence the gene expression. To further determine whether DNMT1 directly interacts with SUV39H1, we examined the physical interaction between the two. We overexpressed *Suv39h1* and *Dnmt1* in HEK293 cells and conducted co-immunoprecipitation assays, in which DNMT1 was first immunoprecipitated and followed by immunoblotting with an antibody against SUV39H1. We found that immunoprecipitation of DNMT1 with a DNMT1 antibody pulled down SUV39H1 protein (Figure 8(c)). We showed previously that the *Wnt10a* promoter is enriched with CpG sites that are subject to DNA methylation [13,14]. We reasoned that if *Suv39h1* deficiency recruits less DNMT1 to the *Wnt10a* promoter as shown in Figure 8(a), the methylation levels at the *Wnt10* promoter would be decreased. Indeed, using pyrosequencing analysis, we found that DNA methylation rates were decreased on at least 16 CpG sites at the *Wnt10a* promoter in *Suv39h1*-deficient L1 adipocytes (Figure 8(d)).

## Discussion

In this study, we demonstrate that the histone methyltransferases *Suv39h1/h2* plays a key role in the regulation of adipogenesis. Our data reveal a unique expression pattern of *Suv39h1* and *Suv39h2*, both of which display a transient spike of expression at the early stage of differentiation, suggesting an important role of these two methyltransferases in the regulation of adipogenesis. Further gain- or loss-of-function studies demonstrate that *Suv39h1* is both necessary and sufficient for 3T3-L1 preadipocytes to achieve complete adipogenesis. We discover that inactivation of *Suv39h1* inhibits adipogenesis while overexpression of *Suv39h1* promotes adipogenesis. During the course of searching for the mechanism underlying *Suv39h*’s effect in adipogenesis, we primarily focused on anti-adipogenic factors based on the two plausible arguments. First, *Suv39h1* and *Suv39h2* are positive regulators of adipogenesis. Second, *Suv39h1* and *Suv39h2* are histone methyltransferases that preferentially catalyse the formation of trimethylation of H3K9 (H3K9me3), a transcriptional repressive mark, increased levels of which must down-regulate anti-adipogenic factors to make *Suv39h* as a positive regulator of adipogenesis. We therefore surveyed the expression of a number of anti-adipogenic factors in both *Suv39h1* knockdown and overexpression L1 cells in a hope that genes with the reciprocal changes in their expression can be converged and identified. Three anti-adipogenic factors that stand out among all the factors we measured are *Wnt10a, Wnt10b and Wnt6*, whose expression is significantly up-regulated in *Suv39h1* knockdown cells while reciprocally down-regulated in *Suv39h1*-overexpressing cells. Although both *Wnt10a* and *Wnt10b* are involved in repression of adipogenesis [25], we [13,14] and others [26,27] have shown that epigenetic regulation of *Wnt10a* expression by both promoter DNA methylation and histone lysine methylation plays a key role in adipogenesis. We therefore focused on *Wnt10a* as a key mediator of *Suv39h1*’s action in adipogenesis. Indeed, *Wnt10a* appears to be required for *Suv39h1*’s effect since *Wnt10a* knockdown largely prevented the inhibitory effect of *Suv39h1* deficiency in adipogenic gene expression. Mechanistically SUV39H1 may repress *Wnt10a* expression via increasing H3K9me3 at its promoter. Interestingly, regulation of *Wnt10* by *Suv39h1* may involve dual epigenetic events in both H3K9 methylation and DNA methylation, cooperation of which may collectively regulate its gene expression. In addition to catalysing H3K9 methylation to repress *Wnt10a* expression, SUV39H1 may also recruit DNMT1 to the promoter of *Wnt10a* to act on DNA methylation, reinforcing the gene silencing. The dual effects of increasing H3K9me3 and DNA methylation at the *Wnt10a* promoter by *Suv39h1* may therefore repress *Wnt10a* expression. Although our study reveals *Wnt10a* as a downstream mediator of *Suv39h1*, it would be interesting to further investigate whether *Suv39h* also acts on H3K9 methylation at other genes’ promoters to regulate their expression during adipogenesis. Of note, *Wnt10a* is a dominant negative regulator of adipogenesis [25], in which many upstream signals are converged. Knockdown of *Wnt10* per se might be sufficient to promote adipocyte differentiation regardless of *Suv39h1* status. Unbiased approaches such as ChIP sequencing with antibodies against SUV39H or H3K9me3 are still warranted to identify genes that *Suv39h* regulates during adipogenesis. It is noteworthy that the observations derived from our study utilizing the murine cell line 3T3-L1 fibroblast may only be restricted to 3T3-L1 adipogenesis and may not be generalized to other cell models and adipogenesis in human. For one, there exists a stark difference in adipogenic process and its regulation between murine and human cell models [28]. Secondly, it is not clear how much the study of adipogenesis using in vitro cell models can recapitulate the real adipogenesis in vivo in the embryonic and post-natal development of adipose tissue and in the development of obesity.

While much of the effort has been devoted to the dissection of transcriptional pathways that control adipogenesis, much less is known about the role of epigenetic regulation in this process. Epigenetic regulation, including histone and DNA methylation, links environmental factors (e.g. diets) to obesity [8,9] where adipogenesis figures in prominently. Emerging evidence has suggested a pivotal role of epigenetic regulation in adipogenic process [27,29]. Extensive epigenetic analysis by Evan Rosen’s lab has revealed a dynamic change of histone marks including both methylation and acetylation spanning across thousands of putative cis-elements during the course of adipogenesis [28], suggesting an important role of the epigenetic events in the regulation of adipocyte differentiation. It is conceivable that changes of these epigenetic events may result in organization of the chromatin structure whereby the transcriptional machinery can subsequently act on specific promoters. For instance, enhanced H3K4me3, a transcriptional active histone mark, has been observed at the *Pparγ* promoter, indicating an active transcription of this master regulator [27]. In contrast, reducing the histone repressive mark H3K27me3 at the *Wnt* gene promoter by demethylation promotes Wnt expression, leading to down-regulation of adipogenesis [27]. We recently found a transient induction of *Dnmt1* at the early stage of 3T3-L1 adipogenesis, suggesting that like SUV39H, DNMT1 may also serve as a pro-adipogenic factor that acts through silencing *Wnt10a* by promoter methylation [14]. Our current study further discloses a crosstalk between SUV39H1 and DNMT1 in regulating Wnt10a expression through coordinated control of H3K9 methylation and promoter DNA methylation. However, a previous report brought us an attention that *Suv39h1*, whose expression was down-regulated during the 3T3-L1 adipogenic process, acted as a negative regulator of 3T3-L1 adipogenesis via inhibiting *Cebpα* [30]. The reason for the discrepancy between this paper’s observation and ours is not clear. We instead found a transient surge of *Suv39h1* expression at the early stage of differentiation, which was absent from their study. Our mRNA data is consistent with the published Affymetrix Array data [28], which profiles the gene expression during the 3T3-L1 adipogenic time course. From the array datasets Mikkelsen et al. deposited in GEO (Accession: GSE20696), we analysed the expression pattern of *Suv39h1* and *Suv39h2* during 3T3-L1 adipogenic process using the NCBI GEO online software GEO2 R and found an expression peak of both *Suv39h1* and *Suv39h2* at day2 of post-differentiation, which mostly corroborated our data (they did not sample at earlier time points within day 2). The rapid induction of *Suv39h* expression at the early stage of differentiation, which represents a critical time window to usher the late transcriptional activation of adipogenesis [20], may suggest *Suv39h* as a positive regulator of adipogenesis at the early stage of differentiation; otherwise, the early rise of *Suv39h* as a negative regulator would shut down the ensuing adipogenic events. With this in mind, *Suv39h1* may exert a biphasic regulatory role in adipogenesis, promoting differentiation at the early stage of differentiation through inhibiting *Wnt10a* while suppressing adipogenesis via inhibiting *Cebpα* at the late stage. This scenario might be analogous to what we have previously reported on *Dnmt1*, another epigenetic silencer playing a dual role: acting as a positive regulator at the early stage of adipogenesis while a negative regulator at the late stage [14]. Indeed, *Suv39h* and *Dnmt1* share a striking similarity in expression pattern featuring an early surge during the 3T3-L1 adipogenic process [14] and are working partners in suppressing *Wnt10a* in the present study. It would be interesting to know whether SUV39H1 and DNMT1 interact to inhibit *Cebpα* at the late stage of adipogenesis, a transcriptional factor responsible for the adipocyte phenotype and function such as insulin sensitivity at the terminal stage of differentiation [31]. Technically, the discrepancy on the effect of *Suv39h1* on adipogenesis may also stem from the difference in *Suv39h* knockdown regimen between the two studies. While they chose to simultaneously knock down both *Suv39h1* and *Suv39h2*, we knocked down *Suv39h1* or *Suv39h2* individually without interfering each other’s expression. Moreover, while they knocked down Suv39h transiently with lentiviral infection, we established L1 knockdown cell lines with antibiotics (puromycin) selection that led to a better knockdown efficiency. However, it is still not clear whether different knockdown approach lead to the discrepancy on the role of *Suv39h* in 3T3-L1 adipogenesis. Future studies are warranted to further examine the role of *Suv39h* in adipogenesis using other cell models originated from both rodents and humans and to validate the observations in vivo using genetic models.

In summary, we find that *Suv39h1* and *Suv39h2* are both rapidly induced at the early stage of differentiation in 3T3-L1 preadipocytes. Our gain-or-loss of function studies show that *Suv39h1/h2* deficiency inhibits 3T3-L1 adipogenesis, while *Suv39h1* overexpression promotes adipocyte differentiation. Upregulation of adipogenesis by *Suv39h1* is likely mediated by down-regulation of the anti-adipogenic factor *Wnt10a*. SUV39 H1 suppresses *Wnt10a* expression via increasing the histone repressive mark H3K9me3 and DNA methylation at its promoter, the latter of which might be mediated through recruiting DNMT1. *Wnt10a* knockdown largely prevents the down-regulation of adipogenesis by *Suv39h1* deficiency. We conclude that *Suv39h1* is a positive regulator of adipogenesis via suppressing the anti-adipogenic factor Wnt10a expression.

## Supplementary Material

Supplemental Material
