# Supplementary material for: The histone methyltransferase Suv39h regulates 3T3-L1 adipogenesis
Source: Adipocyte. 2020 Jul 22;9(1):401–14. doi: 10.1080/21623945.2020.1795422 (PMC7469483; doi:10.1080/21623945.2020.1795422)
Supplement: Supplemental Material [file KADI_A_1795422_SM4436.pdf]

Supplemental figure legends

Supplemental figure 1. The expression profile of the genes encoding histone methyltransferases and demethylases responsible for methylation of H3K9. 3T3-L1 preadipocytes were induced to differentiation and cells were harvested at the time point indicated. Four mRNA samples were pooled at each time point for quantitative PCR analysis of each gene indicated in the figure.

Supplemental figure 2. Quantitative PCR analysis of *Suv39h1* and *Suv39h2* mRNA in *Suv39h* knockdown L1 cells. Gene expression was measured by quantitative PCR. All data are expressed as mean ± SEM, n=4. $*p<0.05$ vs. scramble control.

Supplemental figure 3. Cycle threshold (Ct) values of *Suv39h1* and *Suv39h2* mRNA in L1 preadipocytes. Ct was measured by quantitative PCR using 20ng total RNA/sample. All data are expressed as mean ± SEM, n=6.

Supplemental figure 4. Lentiviral overexpression of *Suv39h1* in 3T3-L1 cells. *Suv39h1* mRNA and protein were measured by quantitative PCR and immunoblotting, respectively. All data are expressed as mean ± SEM, n=4. $*p<0.05$ vs. control.

Supplemental figure 5. *Wnt10a* knockdown in *Suv39h1* knockdown preadipocytes using siRNA. *Wnt10* mRNA (left panel) and protein (right panel) were measured by quantitative PCR and immunoblotting respectively. All data are expressed as mean ± SEM, n=4. $*p<0.05$ vs. scramble control.

Supplemental figure 1

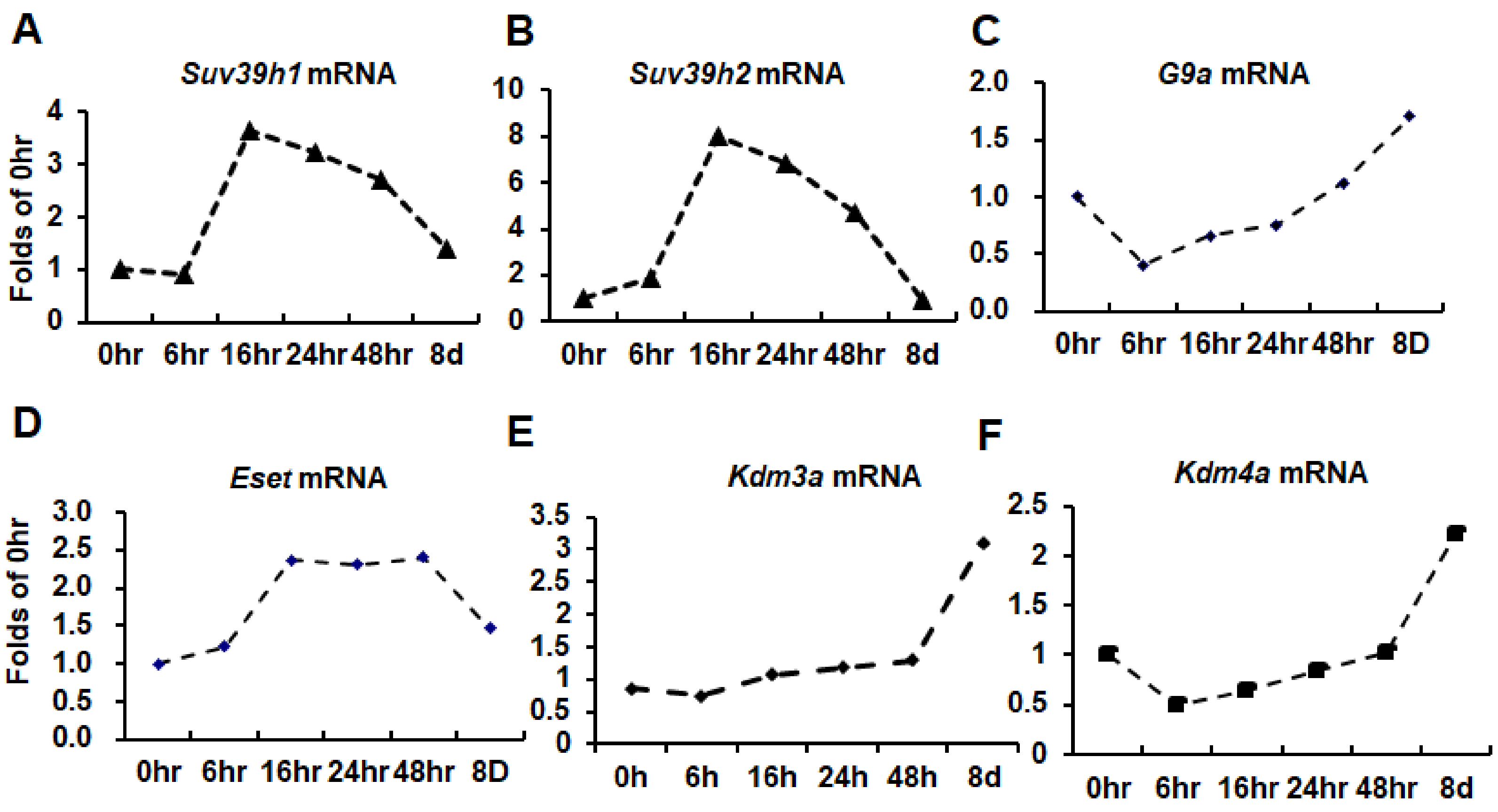

Supplemental figure 2

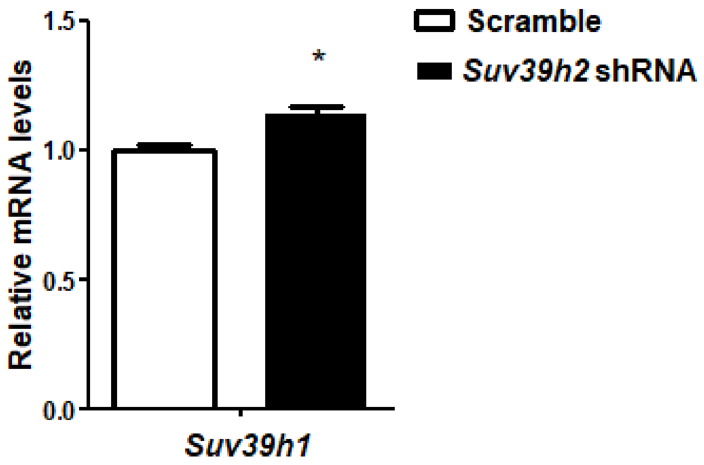

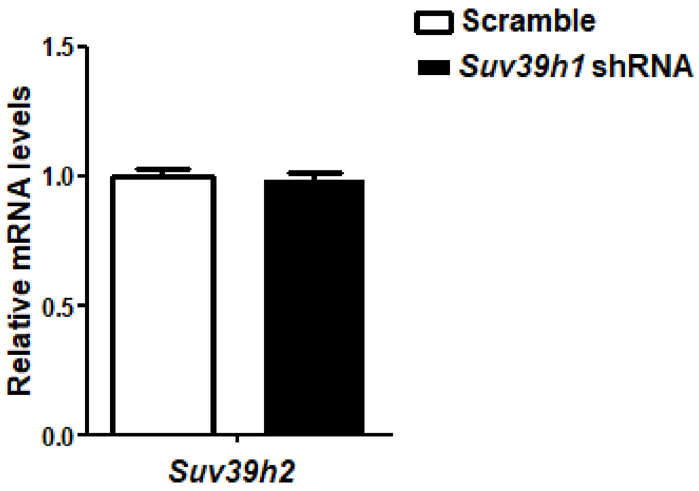

Supplemental figure 3

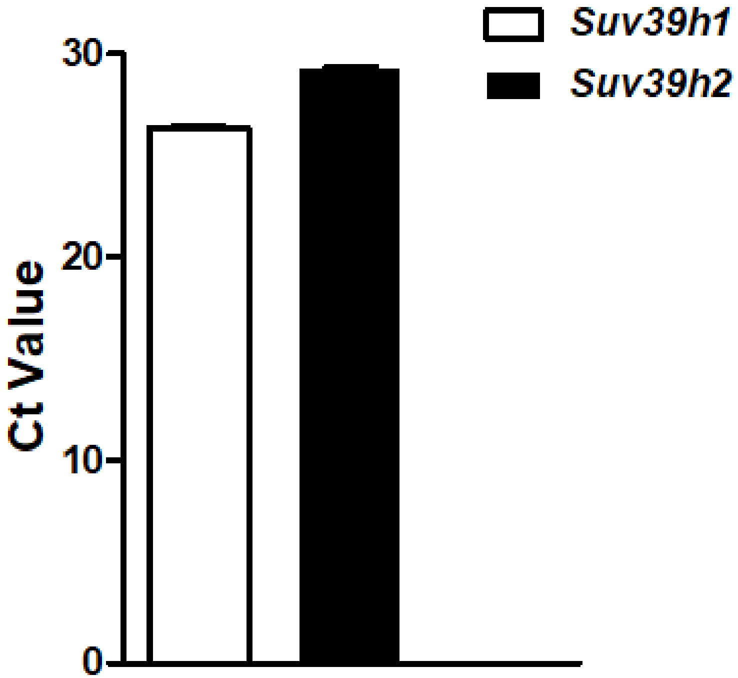

Supplemental figure 4

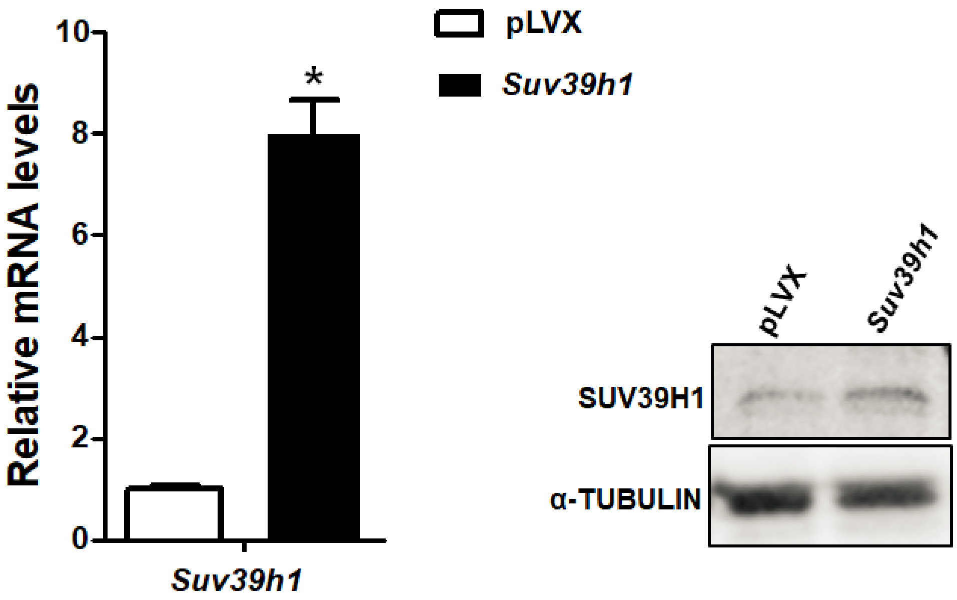

Supplemental figure 5

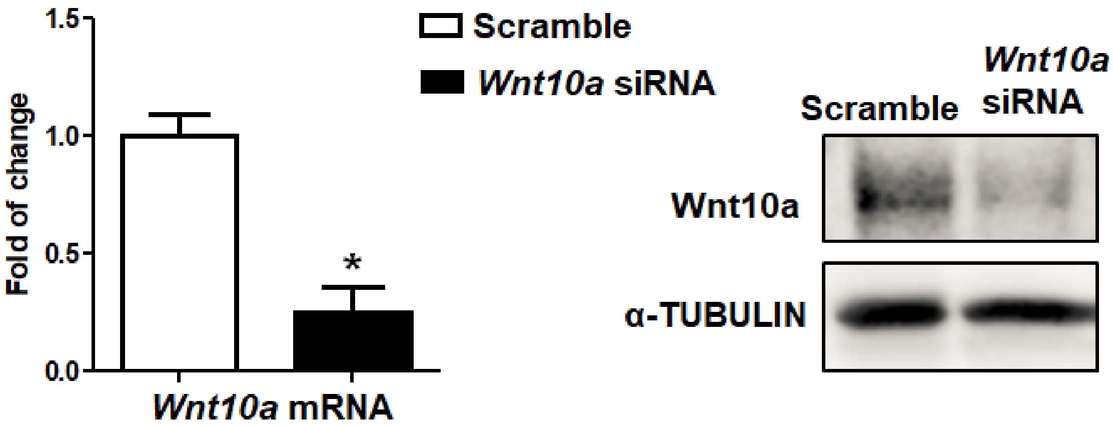

Supplemental Table 1. Amplification and Sequencing primers for Wnt10a promoter pyrosequencing

Primers	Sequences
Amplification primer 1: Forward	5’- GGTGGGGTTGGGTTAGATAGT-3’
Amplification primer 1: Reverse	5’- ACTACCACACACAACTCCTACTCA-3’
Amplification primer 2: Forward	5’- AGGGGTTTTTGGGAAATTTTTTG-3’
Amplification primer 2: Reverse	5’- ACTACCACACACAACTCCTACTCA-3’
Amplification primer 3: Forward	5’- TGTTGAGGGAGTTGTGATTTGAGTAG-3’
Amplification primer 3: Reverse	5’- CCTAAAAAATCCTAACTCTCCAAAAAAAC-3’
Sequencing primer 1	5’- GGTTGGGTTAGATAGTAT-3’
Sequencing primer 2	5’- GTTGGGGTGGGGGGT-3’
Sequencing primer 3	5’- ATTTTTGTGTTAGGAGG-3’
Sequencing primer 4	5’-ACTCCTACTCAAATCAC-3’
Sequencing primer 5	5’- TGTGATTTGAGTAGGAG-3’
Sequencing primer 6	5’-AGTTTTTTATTTTTGGTTTGTT-3’
